# Supplementary material for: DNA sequence classification for diabetes mellitus using NuSVC and XGBoost: A comparative
Source: PLoS One. 2025 Jul 18;20(7):e0328253. doi: 10.1371/journal.pone.0328253 (PMC12273983; doi:10.1371/journal.pone.0328253)
Supplement: Attachment — Submitted filename: Response to reviewers.docx [file pone.0328253.s002.docx]

**Original Manuscript ID:** PONE-D-25-08630

**Original Article Title:** DNA Sequence Classification for Diabetes Mellitus using NuSVC and XGBoost A Comparative

**To:** PLOS ONE

**Re:** Response to reviewers

Dear Editor,

The authors are really very grateful to the Journal Editor efforts in essentially discussing the paper reviews with the reviewers and identifying the further significant points for enhancements. The authors would also like to sincerely thank the reviewers for their valuable remarks and careful feedback which helped them to significantly enhance this work and its presentation. Regardless of the final outcome, the authors sincerely thank the editor and reviewers for supporting them. The productive and valuable remarks enable them to update many parts of the paper as shown by the responses to each comment. Besides, all the updated parts in the manuscript were highlighted in yellow color in order to be easily tracked by the editor and reviewers.

Thank you again for allowing a resubmission of our manuscript, with an opportunity to address the reviewers’ comments.

We are uploading (a) our point-by-point response to the comments (below) (response to reviewers, under “Author’s Response Files*”*), (b) an updated manuscript with yellow highlighting indicating changes (as “Highlighted PDF*”*), and (c) a clean updated manuscript without highlights (“Main Manuscript”*).*

Best regards,

Salloum et al.

| **Authors’ Response to Editor comments:** | | |
| --- | --- | --- |
| **#** | **Reviewers’ comments** | **Authors’ response** |
| **1** | The manuscript is well written and caters to an Important Research Area in the domain of gnetic basis of diseases. However, the manuscript provides very little coverage of literature, does not describe dataset in detail. The authors are advised to provide more literature support and more insight in to the dataset. Also the comparison of only two machine learning algorithms does not fully validate the effectiveness of learning from the DNA sequence data. The authors are advised to compare their models with other established models using similar type of data. | *Thank you for your insightful comment. In response, we have extensively revised and expanded the Related Work section by integrating a broader and more recent set of studies focused on machine learning and deep learning applications in genomic-based diabetes prediction. To improve clarity and coherence, we restructured the section into five thematic subsections: 2.1 Classical Machine Learning Models and Feature Selection in Genomics, 2.2 Ensemble Learning for Predictive Modeling of Genetic Risk, 2.3 Deep Learning Approaches for DNA and SNP Sequence Classification, 2.4 Natural Language Processing Techniques and Data Imbalance Solutions, and 2.5 Current Gaps and Study Contribution. Each subsection presents a targeted synthesis of relevant literature, including dataset sources, feature types, modeling approaches, and key performance metrics. We also updated the dataset description with detailed characteristics and clarified our model evaluation by comparing our results with a broader set of established models reported in the literature. This restructuring directly addresses the concerns regarding limited literature coverage, dataset detail, and model comparison.* |
| **2** | The data cleaning process is incompletely covered and should describe the process in detail. The authors are advised to include a graphical view of the model architecture to make the manuscript more intuitive. | *Thank you for highlighting the need to elaborate on the data cleaning and preprocessing process and to include a visual representation of the model architecture. In response, we have substantially revised Section 3.2 to provide a detailed and transparent description of each preprocessing step. This includes how the DNA sequences were cleaned using BioPython to eliminate ambiguous nucleotides, the binary labeling procedure, TF-IDF-based feature encoding using 3–6 character n-grams, the use of SMOTE to address class imbalance, and the application of stratified 80/20 data splitting to preserve class distribution across training and validation sets.*  *Additionally, we have added a graphical diagram illustrating the full model architecture and processing pipeline, from raw FASTA input to final classification using NuSVC and XGBoost. This visualization enhances clarity and helps readers intuitively follow the workflow. These updates aim to improve the manuscript’s reproducibility and meet the journal’s standard for methodological transparency.* |

| **Authors’ Response to Reviewer 1:** | | |
| --- | --- | --- |
| **#** | **Reviewers’ comments** | **Authors’ response** |
| **1** | Unjustified use of SMOTE on a balanced dataset | *We appreciated the reviewer’s careful observation regarding the use of SMOTE. We clarified that SMOTE was initially applied during the early stages of model development when minor imbalances appeared after stratified splitting into training and validation sets. Although the overall dataset was balanced, small variations at the training subset level could still introduce bias, especially for models sensitive to class distributions. To address the reviewer’s concern, we reevaluated the model without SMOTE and confirmed that XGBoost and NuSVC maintained similar performance trends, with only marginal changes in precision and recall. We revised the manuscript to reflect this clarification and to avoid any impression of unjustified preprocessing steps.* |
| **2** | The dataset is initially balanced (1,296 sequences per class). Applying SMOTE here is unnecessary and may introduce artificial patterns. Please clarify the class distribution after train/validation split and justify the need for oversampling. | *We thanked the reviewer for raising this important point. We acknowledged that the original dataset contained 1,296 sequences per class, making it balanced. However, after performing an 80/20 stratified train-validation split, slight imbalances appeared within the training set. Specifically, the training data contained 1,036 diabetic and 1,030 non-diabetic sequences. Although the imbalance was minor, we initially applied SMOTE as a precaution to ensure uniformity for both classes during model training. We recognized that applying SMOTE to an already balanced or near-balanced dataset could introduce artificial patterns. In response, we retrained the models without SMOTE and observed that the performance metrics remained consistent, confirming that SMOTE had no significant impact. We updated the manuscript to clarify this point and removed unnecessary references to SMOTE where appropriate.* |
| **3** | Lack of robust model validation | *We appreciated the reviewer’s comment regarding the robustness of model validation. We initially validated the models using a simple train-validation split, which may not have fully captured variability across different subsets. To strengthen the validation, we restructured the experimental design and applied 5-fold cross-validation across the training data. We reported the mean and standard deviation for all key metrics, including accuracy, precision, recall, and F1-score. This approach provided a more reliable assessment of the models' generalization ability. We updated the manuscript to include these new validation results and emphasized the improved robustness of the evaluation process.* |
| **4** | Results are presented for a single 80–20 split. No k-fold cross‐validation or external test set is used, raising concerns about overfitting—especially given the perfect AUC of 1.00 for XGBoost. Please include repeated cross‐validation (e.g., 5- or 10-fold) and report mean ± SD for each metric | *Initially, we presented results based on a single 80–20 split, which limited the robustness of our evaluation. To address this concern, we implemented 5-fold cross-validation and repeated the experiments. We computed and reported the mean and standard deviation (mean ± SD) for accuracy, precision, recall, F1-score, and ROC-AUC. This provided a more reliable estimate of model performance and mitigated the risk of overfitting. We found that the XGBoost model maintained high performance with a mean AUC close to 0.99 ± 0.01, confirming its strong but realistic classification ability. We updated the Results and Methodology sections accordingly to reflect these changes.* |
| **5** | Missing statistical significance testing | *We thank the reviewer for highlighting this point. We have now included paired t-tests comparing NuSVC and XGBoost across 10-fold cross-validation. The results confirm that XGBoost’s improvements in accuracy and other metrics are statistically significant (p < 0.05), and we have added this analysis to the Results section.* |
| **6** | Differences in accuracy (98% vs 87%) and AUC (1.00 vs 0.95) require statistical validation (e.g., DeLong’s test for ROC curves). Report p-values or confidence intervals to demonstrate that XGBoost’s superiority is not due to chance | *We appreciate the reviewer’s suggestion. In response, we performed statistical validation using paired t-tests for accuracy and DeLong’s test for AUC scores. The results showed that the differences between XGBoost (98%, AUC = 1.00) and NuSVC (87%, AUC = 0.95) are statistically significant (p < 0.05). These findings, including p-values and confidence intervals, have been added to the Results section to support the robustness of the performance comparison.* |
| **7** | Insufficient hyperparameter and feature‐engineering detail. | *We thank the reviewer for the comment. In response, we revised the Feature Extraction section to include detailed explanations of the k-mer strategy and TF-IDF parameters used, clearly describing how DNA sequences were transformed into meaningful numerical features for classification.* |
| **8** | The choice of k-mer sizes (k = 3 and 6) and TF-IDF parameters is not justified. Details of grid search (parameter ranges, scoring metric) are omitted. Please provide a complete description of all hyperparameters and preprocessing settings in either the Methods or a Supplementary Table. | *We thank the reviewer for the comment. We have now justified the choice of k-mer sizes (3–6) and TF-IDF parameters in the revised Methods section. We also added a detailed description of the grid search process, including parameter ranges and ROC-AUC as the scoring metric.* |
| **9** | Misuse of terminology | *We thank the reviewer for noting the misuse of terminology. We reviewed and corrected all inaccurate terms throughout the manuscript to ensure consistency with standard machine learning and genomics language.* |
| **10** | References to “training over 10 epochs” for XGBoost and NuSVC are incorrect: these models do not train in epochs. Replace with precise descriptions (e.g., number of boosting rounds for XGBoost, number of support vectors or CV folds for NuSVC). | *We thank the reviewer for the clarification. We removed all incorrect references to "epochs" and replaced them with precise descriptions: XGBoost was trained with up to 300 boosting rounds, and NuSVC was evaluated using 10-fold cross-validation.* |
| **11** | Overly optimistic performance claims. | *We thank the reviewer for this important observation. In response, we revised the language throughout the manuscript to avoid overstatement and to more cautiously interpret the model performance. We now emphasize the limitations of the dataset, note potential overfitting risks, and clarify that while XGBoost performed well on our dataset, further validation on independent and larger genomic datasets is needed to confirm its generalizability.* |
